# Bringing Rights of Older Persons to the Real World: Lessons Learned From a Rights-Based Training

**DOI:** 10.1093/geroni/igab046.1316

**Published:** 2021-12-17

**Authors:** Kimberly Stoeckel, Kelly Fitzgerald

**Affiliations:** 1 UMBC Erickson School of Aging Studies, Baltimore, Maryland, United States; 2 University of Maryland Baltimore County, Baltimore, Maryland, United States

## Abstract

When gerontological education and training are grounded in a rights-based approach, this framing provides a tool to ensure the rights of older people are advocated for and experienced. A “train the trainer” program was given to non-governmental organizations (NGOs) providing services to refugees in Jordan. The goal of the training was to educate NGOs on how to use a rights-based approach when responding to and supporting older refugees. The training covered a range of topics in ensuring the rights of older people in the provision of care, protection, and inclusion. Training outcomes revealed an increased awareness of the rights of older people. Skills and knowledge gained as a result of this training empowered participants to further develop their own work, within their cultural context, to reflect a rights-based approach to services and programs.

